# Editorial for Special Issue “Role of Microorganisms and Their Metabolites in Agriculture, Food and the Environment”

**DOI:** 10.3390/ijms252111417

**Published:** 2024-10-24

**Authors:** Barbara Sokołowska

**Affiliations:** Department of Microbiology, Prof. Wacław Dąbrowski Institute of Agricultural and Food Biotechnology—State Research Institute, Rakowiecka 36 St, 02-532 Warsaw, Poland; barbara.sokolowska@ibprs.pl

Microorganisms and their metabolites play a crucial role in agriculture, food production, and environmental sustainability, offering solutions to challenges like soil degradation, pest management, and climate resilience. Their applications span biocontrol, nutrient cycling, and the enhancement of plant growth. In agriculture, beneficial microbes such as plant growth-promoting rhizobacteria (PGPR) and arbuscular mycorrhizal fungi have been recognized for improving soil fertility and crop resilience. These microbes facilitate nutrient uptake, enhance nitrogen fixation, and promote plant growth through various mechanisms, including hormone production and disease resistance induction [1]. Their ability to form symbiotic relationships with plant roots helps in sustaining agricultural productivity, especially under drought or high-salinity stress [2].

Microbial metabolites, including antibiotics, enzymes, and volatile organic compounds (VOCs), have emerged as potent biocontrol agents, providing an eco-friendly alternative to chemical pesticides. These metabolites suppress plant pathogens such as fungi and bacteria, reducing the reliance on synthetic agrochemicals [3]. Notably, bacteria like Bacillus and Pseudomonas have demonstrated effectiveness in managing soilborne diseases, contributing to sustainable pest management practices [4].

In the context of food production, microbial fermentation has been pivotal in extending the shelf-life and safety of various food products. Lactic acid bacteria, for instance, play a significant role in the fermentation of dairy, vegetables, and cereals, enhancing their nutritional profiles and preventing spoilage [5] Biopreservation based on the use of LAB and their metabolites may be associated with an increase in food safety as well as other benefits for human health, considering their ability to improve nutritional value by producing some vitamins, organic acids and other compounds. LAB show antibacterial and antifungal activity. In addition, LAB may detoxify second metabolites of filamentous fungi (mycotoxins) using different mechanisms, including bioabsorption, biotransformation and bioadhesion. This natural preservation method reduces food waste and minimizes the need for chemical preservatives, aligning with consumer demand for cleaner, more sustainable food production methods [6].

Environmental applications of microorganisms extend to bioremediation, a process in which certain microbial species are harnessed to degrade pollutants and restore contaminated soils and water. This is particularly of interest in areas affected by industrial activities, where microbes can convert toxic compounds into less harmful substances, improving soil health and ecosystem resilience [7].

Recent advances in microbiome research, including next-generation sequencing and genome editing, have provided deeper insights into the complex interactions between microbes and their environments. These tools have enabled scientists to design microbial consortia that improve soil health, enhance plant productivity, and ensure the sustainable use of agricultural inputs [1].

As research progresses, the potential to engineer microbial communities tailored to specific agricultural or environmental needs continues to grow, promising a more sustainable future for food systems and ecosystem management [8].

In general, the role of microorganisms and their metabolites is pivotal in advancing sustainable agricultural practices, improving food safety, and contributing to environmental restoration efforts. Their integration into modern practices offers a viable pathway toward achieving global food security while mitigating the environmental impact of conventional agricultural methods.

Despite significant advancements in the understanding of microorganisms and their applications, several gaps in knowledge remain that limit the broader implementation of microbial solutions in agriculture, food production, and environmental management. Issues such as understanding the complexity of microbial interactions within ecosystems, the potential for optimizing microbial consortia for specific biotechnological applications, and the full spectrum of their roles in biocontrol and bioremediation remain unexplored. Recent studies have sought to fill these gaps, providing new insights into the intricate roles of microbes and their metabolites across various applications.

One major area with limited data available is the interaction between microbial communities and plant pathogens, particularly how these interactions can be leveraged for biocontrol. Previous research has primarily focused on individual microbial strains or simple microbial consortia, often failing to capture the complexity of natural microbial interactions. In the first contribution, Duan et al. (2024) address this by exploring the dynamics of Enterobacteriaceae within the Asian citrus psyllid (*Diaphorina citri*), a vector for the devastating Huanglongbing (HLB) disease in citrus plants. Their study reveals that HLB infection alters the microbial community composition, particularly increasing the abundance of Enterobacteriaceae. This finding highlights a potential biomarker for disease detection and suggests that manipulating microbial communities could play a role in disease management. By identifying specific microbial taxa associated with HLB, this research provides insights into the microbial dynamics that could be further explored for biocontrol strategies in agricultural settings.

Another significant challenge lies in the biodegradation of persistent organic pollutants, which is crucial for environmental restoration but is often limited by the slow degradation rates of such compounds. Dong et al. (2023) (Contribution 2) provide valuable insights into this area by demonstrating the complete mineralization of benzo(a)pyrene, a highly toxic polycyclic aromatic hydrocarbon, using a genetically modified strain of *Pseudomonas benzopyrenica* BaP3. Their study not only identifies specific genetic pathways that enhance biodegradation but also underscores the potential of using recombinant microbes for the remediation of contaminated sites. The ability to optimize such strains for targeted bioremediation represents a critical advancement toward reducing environmental pollution, particularly in soils affected by industrial activities.

The production of value-added metabolites from agricultural byproducts remains an underexplored area, despite its potential to add economic value to waste streams. In a study on the production of gamma-decalactone—a valuable aroma compound—by *Yarrowia lipolytica* (CContribution 3), Małajowicz et al. (2023) explore the use of water treated with plasma to enhance metabolite production. By showing that plasma-treated water can improve both the growth of *Y. lipolytica* and the yield of gamma-decalactone, this research suggests a novel approach to enhance microbial fermentation processes. This finding fills a gap in the knowledge regarding the optimization of microbial metabolite production under non-traditional conditions, potentially reducing the costs and environmental footprint of biotechnological processes.

Furthermore, the role of complex microbial interactions in improving soil health and plant resilience is an area where knowledge is still emerging. Although the benefits of arbuscular mycorrhizal fungi and plant growth-promoting rhizobacteria are well documented, less is known about how microbial consortia function under varying environmental conditions. In the research on microbial consortia and their role in protecting plants against pathogens (Contribution 4), Maciag et al. (2023) highlight how multi-species microbial formulations can enhance disease resistance and improve plant resilience. This study demonstrates that combining different microbial species can result in more robust biocontrol solutions, addressing the variability and unpredictability often encountered in field conditions. By providing evidence that consortia are more effective than single strains in protecting plants, their research points to a promising direction for future research in sustainable agriculture.

Another emerging field is the use of microorganisms for the valorization of agro-industrial waste. Zhang et al. (2023) (Contribution 5) investigate the utilization of buckwheat hulls as a source of fermentable dietary fiber to address a key gap in developing sustainable food systems. Their study demonstrates that bioprocessing can enhance the bioavailability of phenolic compounds in buckwheat hulls, thus turning agricultural waste into a source of valuable bioactive compounds. This research aligns with the broader goals of the circular economy in agriculture by highlighting the conversion of waste into high-value products. In so doing, it enhances our understanding of the mechanisms underlying the microbial transformation of waste products, thus paving the way for more efficient and environmentally friendly approaches to food production.

The field of microbial genomics has provided new tools for understanding and optimizing microbial functions; however, the practical applications of these tools in natural and agricultural settings are still being developed. Hu et al. (2023) (Contribution 6) showcase the genomic analysis of *Penicillium oxalicum* as an example of how genomic insights can be translated into practical applications. By identifying enzymes involved in lignocellulose degradation, this study contributes to the development of microbial strains that can efficiently break down plant biomass, which is crucial for biofuel production and the utilization of agricultural residues. Their research highlights the potential of microbial genomics to identify novel enzymes and pathways that can be harnessed for industrial applications, addressing the need for renewable energy sources and sustainable waste management.

Understanding the role of microorganisms in natural nutrient cycling is another area of ongoing research. In a study on microbial transformations of manganese-containing minerals, Farkas et al. (2023) (Contribution 7) highlight the role of microorganisms in the cycling of critical minerals, which is crucial for soil health and fertility. This research provides foundational knowledge for using microbial processes in soil remediation, helping to maintain ecosystem stability and productivity.

Research into microbial fermentation has also been pivotal for food preservation and safety; nevertheless, process optimization is still needed for enhanced outcomes. In their study on *Kluyveromyces marxianus* and its bioconversion capabilities, Drężek et al. (2023) (Contribution 8) explore how microbial fermentation can process whey permeate into valuable bioproducts. Their research addresses waste reduction and contributes to sustainable food production by transforming byproducts into beneficial substances like ethanol.

Although the development of microbial genetic editing tools holds promise for refining microbial strains tailored for specific purposes, practical applications remain a challenge. Using CRISPR/*Cas9* in *Cupriavidus nantongensis*, Zhang et al. (2023) (Contribution 9) demonstrate how targeted genetic modifications can enhance the metabolic capabilities of industrially relevant strains. This approach is crucial for developing microbial solutions that can adapt to different environmental conditions and improve their efficiency in industrial processes.

Finally, the use of bacteriophages as biocontrol agents in food safety presents a promising alternative to chemical interventions; however, their application in complex food systems requires further validation. In the last contribution, Wójcicki et al. (2023) provide a practical example of this approach by investigating bacteriophages targeting *Salmonella* in food matrices. Their findings indicate that specific bacteriophages can significantly reduce *Salmonella* contamination in ready-to-eat foods, offering an effective and natural method for improving food safety. By addressing the challenges of phage stability and specificity, this research demonstrates a viable biocontrol method for reducing foodborne pathogens.

The studies amassed in this Special Issue highlight significant progress in leveraging microorganisms for agriculture, food, and environmental applications. They address critical gaps in understanding microbial interactions, biodegradation pathways, and the optimization of microbial processes for practical applications. However, further research is needed in several areas, including the large-scale application of engineered microbial strains, the long-term environmental impacts of microbial amendments, and the development of microbial solutions tailored to specific regional challenges. Future research should also explore the integration of next-generation sequencing and synthetic biology tools to design microbial consortia that are both efficient and resilient in varied environmental conditions. By advancing our understanding in these areas, the potential of microorganisms as sustainable contributors to food security and environmental health can be fully realized.
